# Who Fears to Speak?

**Published:** 1918-08-17

**Authors:** 


### A NURSES' CHARTER OF LIBERTY.

A Successful Experiment.

For a long time nothing has afforded me greater pleasure than going through my accumulated mail containing letters and post-cards from nurses expressing their ideal of a Charter of Liberty. This experiment in articulation has been a triumphant success, for, apart altogether from the main object in view, the mere fact of inducing a little multitude of working nurses to express their considered opinions on an economic matter, vitally affecting their lives, is an achievement which, so far as I am aware, is without parallel. My mail corresponds with a large confession album, and its contents afford much food for reflection. When one nurse sends me a post-card, for example, representing forty of her fellow-workers, I cannot resist the conclusion that the writer comes under the category of "agitator" to which I referred last week, and when another writes "We are dumb because we are too tired and crushed to speak," I feel that the floodgates of truth begin to open. My first duty, therefore, is in the most hearty manner to thank one and all of the contributors. I do not exaggerate when I state without reserve that in responding to my invitation each individual writer has done substantial service to the common cause, and I venture to predict that this will become more and more manifest as the weeks pass.

The Worker's Claim.

Some post-cards are voluminous; others brief. Considering that they come from all parts of Great Britain, including the far north of Scotland, and from the London Hospital itself, which apparently has no need for a Charter—from which indeed a Charter for other hospitals fulminated—I am astonished at the unanimity of the wishes, and the warmth with which they are expressed. These I propose analysing and tabulating. But before going to this trouble, and from a casual perusal, it is easy to see that the song of the worker is the same throughout, with variations, some of the variations being both interesting and important. The post-card which in my opinion is by a long way the best, which crystallises the views of the great majority who have written, is also by a long way the briefest. Particularly pleasing, moreover, it is to observe that the writer is the matron of a large and representative metropolitan hospital. So excellently expressed, so reasonable, so comprehensive is this document that I regard it as worthy of being framed and hung up in every hospital board-room in the kingdom. It constitutes in itself a magnificent Charter of Liberty. Here it is :—

**EVERY NURSE SHOULD HAVE:**
**An Eight-hour Day**
**A Day Off Once a Week**
**Including a Night Away from Hospital**
**Four Weeks' Holiday in a Year**

My first thought when I read this post-card was that the writer is an ideal matron. In every word there is a human touch, a note that bespeaks true comradeship and a big mind. I venture to say, without knowing the lady who wrote it, that her hospital is an ideal place for women to work, and for sick men and women to come for healing. The most unwholesome environment for suffering patients is an institution where the nurses are weary and repressed, where they are dragooned so that they can never call their bodies or their souls their own. And I say, furthermore, that if my experiment in articulation had produced naught else save this one post-card, I should regard the harvest as rich.

Who Fears to Speak?

Can anybody point to a defect? Can any object to an eight-hours' day? Can any other community of men or women wage-earners be instanced whose working hours are longer? If so, this is the time to speak. The hospital nurse has been for a long time dumb, but she has opened her mouth, and this is her brief speech. There have been endless and futile arguments about registration, representation, "laymen," limited liability companies, "society women," "gold bugs," and all the rest. We have at last a useful theme, and I want to know who fears to speak. Who is going to be dumb? This Charter, be it observed, is not made in a newspaper office, nor yet is it the Utopian ideal of an impractical and undisciplined "agitator." It is the claim of a very large number of nurses scattered throughout the kingdom, and expressed by one who is herself in charge of nurses and possessed of a full sense of responsibility to patients in hospital. What I desire if possible to ascertain is whether any hospital governor regards the claim as unreasonable, and, if so, why. The criticism I should most highly esteem is Lord Knutsford's. He is the chairman of the largest London hospital, and, as such, he is primarily responsible for having set this argument in motion. So deep, indeed, is his interest in the nurses in general that he stepped outside the ambit of his own institution and prepared what he described as a Charter of Liberty for nurses seeking employment in others. I asked him once before to defend the provisions of his Charter, and he responded. He said that it embodied the irreducible minimum that a nurse should accept. I venture once again to ask him to speak, and on this occasion in criticism of a Nurses' Charter prepared by the worker. The document is not voluminous. It is void of technicalities. An eight-hours' day, one day's rest in seven, and four weeks' leave within a year—nothing more, nothing less. Justice to the nurse is the motto of the chairman of the "London." In the name of justice, therefore, I ask him to express an opinion on the text. There may be a hundred reasons why the rank and file of the nursing profession are dumb. Lord Knutsford is a practised speaker in public and in private. His is the pen of a ready writer. He is a senator in the High Court of Parliament and a public man. What sayeth he? Tens of thousands of women toiling in the cause of the nation's health will await his words.

The Broken Spell.

In the meantime nurses will appreciate from what has transpired the immense value of free speech. They will likewise appreciate the power that lies in union. Remember Jericho! Remember that the walls thereof fell, not because of aircraft or cannonading, but because of the voice of the multitude. "All the people shouted." This expedition into the unknown marks the beginning of a movement that may have consequences of far-reaching importance. The spell of silence is broken. At present it is "just a whisper, nothing more." But it will grow in volume and intensity, so that not alone hospital matrons and hospital boards, but the British nation, will realise that the call of the British nurse must and shall be answered.

IERNE.

